# Supplementary material for: Diagnosis of Acute Leukemia by Multiparameter Flow Cytometry with the Assistance of Artificial Intelligence
Source: Diagnostics (Basel). 2022 Mar 28;12(4):827. doi: 10.3390/diagnostics12040827 (PMC9029950; doi:10.3390/diagnostics12040827)
Supplement: Supplementary file 1 [file diagnostics-12-00827-s001.zip › supplementary Table S1 Cell Category vs Tube Antigen.pdf]

Cell Category vs Tube Antigen

	CD Markers	Common Categories	Subtype Categories
**tube 1**	CD45		
**tube 2**	CD45, CD34, CD117, HLA-DR, CD38	lymph, mono, granu, blast, NRBC	ALL blast, AML blast
**tube 3**	CD45, CD11b, CD13, CD16, CD33, CD34	lymph, mono, granu, blast, NRBC	ALL blast, AML blast
**tube 4**	CD45, CD14, CD15, CD34, CD64	lymph, mono, granu, blast, NRBC	ALL blast, AML blast
**tube 5**	CD45, CD19, CD20, CD5, CD22	lymph, mono, granu, blast, NRBC	B-cell, B-ALL blast
**tube 6**	CD45, CD3, CD4, CD8, CD2, CD7, CD56	lymph, mono, granu, blast, NRBC	T-helper, T-killer, NK, T blast
**tube 7**	CD45, MPO, CD34, cCD3, CD79a	lymph, mono, granu, blast, NRBC	M blast, T blast, B blast
