# Supplementary material for: Diagnosis of Acute Leukemia by Multiparameter Flow Cytometry with the Assistance of Artificial Intelligence
Source: Diagnostics (Basel). 2022 Mar 28;12(4):827. doi: 10.3390/diagnostics12040827 (PMC9029950; doi:10.3390/diagnostics12040827)
Supplement: Supplementary file 1 [file diagnostics-12-00827-s001.zip › supplementary Table S2.pdf]

Abnormal cell proportion of AI and manual (difference > 20%)

casePath	abnor-1_manual	abnor-1_AI	difference
/AML-M3/100005	95.2	73.5	21.7
/AML-M3/100008	83.2	61.8	21.4
/AML-M3/100036	86.8	59.1	27.7
/AML/100060	46.5	79.4	-32.9
/ZSUFHnewdata/100148	95.1	59.9	35.2
/ZSUFHnewdata/100174	58.5	94.2	-35.7
/ZSUFHnewdata/100185	92.3	71.2	21.1
/ZSUFHnewdata/100199	96.2	48.4	47.8
/ZSUFHnewdata/100201	61.8	82.9	-21.1
/ZSUFHnewdata/100213	82.7	53	29.7
/ZSUFHnewdata/100218	61.6	40.8	20.8
/ZSUFHnewdata/100228	93.5	67.8	25.7
